# Migrating curlews on schedule: departure and arrival patterns of a long-distance migrant depend on time and breeding location rather than on wind conditions

**DOI:** 10.1186/s40462-021-00252-y

**Published:** 2021-03-17

**Authors:** Philipp Schwemmer, Moritz Mercker, Klaus Heinrich Vanselow, Pierrick Bocher, Stefan Garthe

**Affiliations:** 1 Research and Technology Centre (FTZ), University of Kiel, Hafentörn 1, 25761 Büsum, Germany; 2 Bionum GmbH – Consultants in Biological Statistics, 21129 Hamburg, Germany; 3 Littoral Environnement et Sociétés Laboratory (LIENSs), University of La Rochelle CNRS, 17000 La Rochelle, France

**Keywords:** Shorebird, Wadden Sea, Flight altitude, Phenology, GPS tracking, Repeatability

## Abstract

**Background:**

Departure decisions in long-distance migratory bird species may depend on favourable weather conditions and beneficial resources at the destination location, overarched by genetic triggers. However, few studies have tried to validate the significance of these three concepts simultaneously, and long-term, high-resolution tagging datasets recording individual movements across consecutive years are scarce. We used such a dataset to explore intraspecific and intra-individual variabilities in departure and arrival decisions from/to wintering grounds in relation to these three different concepts in bird migration.

**Methods:**

We equipped 23 curlews (*Numenius arquata*) wintering in the Wadden Sea with Global Positioning System data loggers to record their spatio-temporal patterns of departure from and arrival at their wintering site, and the first part of their spring migration. We obtained data for 42 migrations over 6 years, with 12 individuals performing repeat migrations in consecutive years. Day of year of departure and arrival was related to 38 meteorological and bird-related predictors using the least absolute shrinkage and selection operator (LASSO) to identify drivers of departure and arrival decisions.

**Results:**

Curlews migrated almost exclusively to Arctic and sub-Arctic Russia for breeding. They left their wintering site mainly during the evening from mid- to late April and returned between the end of June and mid-July. There was no difference in departure times between the sexes. Weather parameters did not impact departure decisions; if departure days coincided with headwind conditions, the birds accounted for this by flying at higher altitudes of up to several kilometres. Curlews breeding further away in areas with late snowmelt departed later. Departures dates varied by only < 4 days in individual curlews tagged over consecutive years.

**Conclusions:**

These results suggest that the trigger for migration in this long-distance migrant is largely independent of weather conditions but is subject to resource availability in breeding areas. The high intra-individual repeatability of departure days among subsequent years and the lack of relationship to weather parameters suggest the importance of genetic triggers in prompting the start of migration. Further insights into the timing of migration in immatures and closely related birds might help to further unravel the genetic mechanisms triggering migration patterns.

**Supplementary Information:**

The online version contains supplementary material available at 10.1186/s40462-021-00252-y.

## Background

Migration is an essential part of the life cycle of a wide range of species, with potentially important consequences for their fitness [1–5]. Birds show the most extensive and far-ranging migrations [6–8]. Careful timing of migration is crucial, particularly in long-distance migrants, and previous work revealed three general concepts affecting the onset of migration in birds. (1) Departure decisions were significantly related to favourable weather conditions during northbound spring and southbound autumn migration, as consistently demonstrated for different groups of birds [9–12]. (2) The start of northbound spring migration needs to coincide with beneficial environmental resources in the destination areas, as a prerequisite to ensuring fitness [13, 14]. In this respect, particularly Arctic and sub-Arctic breeders need to time their migration to arrive in their breeding areas shortly after snowmelt so that breeding efforts coincide with periods of peak food availability for their young [14, 15] (3) Genetic triggers and endogenous programmes also play an overarching role in determining the timing of migration particularly in northbound spring migration [16–18]. The relevance of this last concept may be difficult to prove; however, one possible approach to investigating this concept would be to assess intra-individual variability by examining repeated migration patterns in the same individuals in different years, though this has rarely been achieved using movement data [19–22].

The above three concepts have mainly been studied independently using non-individual approaches, such as visual observations, colour-ringing studies [23, 24], and radar techniques [25, 26]. However, although tagging studies have recently provided some initial insights into individual-based departure decisions with respect to weather conditions (e.g. [12, 27]), tagging studies assessing individual repeatability of departure decisions (required to prove the role of genetic triggers for bird migrations) are still lacking [19].

The current study therefore aimed to assess the relevance of each of the three concepts simultaneously, using a high-resolution tagging dataset based on long-term attachment of Global Positioning System (GPS) devices to birds, which allowed individual migration decisions to be analysed in relation to weather, location of breeding sites, and individual repeatability across consecutive years.

We used the Eurasian Curlew (*Numenius arquata*) as a model long-distance migrating shorebird. We studied departure and arrival patterns at one of the species’ most important nonbreeding sites (i.e. site used outside the migratory period during the boreal winter, hereafter referred to as “wintering”) on the East Atlantic Flyway, the Wadden Sea. Despite strong population decreases in the flyway population as a whole [28], the numbers of curlews wintering in the Wadden Sea have remained stable at around 200,000–260,000 individuals, accounting for around 40% of the total flyway population [29, 30]. However, information on the migration patterns of curlews wintering in the Wadden Sea is scarce, and to our knowledge, Schwemmer et al. [31] presented the only preceding preliminary study on this topic. A previous study from south-west England investigated the arrival and departure patterns of curlews based on a dataset of re-sightings of colour-marked individuals [23]. In the current study, we equipped curlews with GPS data loggers that recorded the times of arrival and departure of each individual bird in the Wadden Sea. This allowed individual departure and arrival patterns to be precisely related to meteorological data and location of the breeding area, and allowed the repeatability of temporal patterns across subsequent years to be assessed. We proposed five hypotheses. (1) Given that tailwinds will increase flight and migration speeds [6, 32, 33], we expected curlews to time their departure from and arrival at their wintering grounds according to favourable wind and weather conditions, especially in relation to tailwind conditions, lack of precipitation, and air temperature, as found in other bird species [12, 25, 27, 34]. (2) In line with this, we expected flight heights (as recorded by GPS tags) to increase during non-tailwind conditions to allow the birds to reach air layers with improved wind conditions [25, 35]. (3) We predicted significant effects of departure date and tailwind component on the distance to and duration of the first stop-over event. Previous studies indicated that headwind conditions could significantly shorten the distance to the first migration stop-over and increase the stop-over duration to allow birds to refuel before continuing (e.g. [36]). (4) We hypothesized that birds breeding further from their wintering site at higher latitudes and more easterly longitudes would depart later to time their arrival at their Arctic and sub-Arctic breeding grounds according to snowmelt and the underlying availability of food resources [13–15]. In this context, we expected males to arrive at the wintering sites later than females, because, as for other shorebird species, females are known to desert their chicks earlier than males [37]. (5) We expected a certain level of repeatability in curlew departure dates (in accordance with [24]), irrespective of weather conditions, because circadian rhythms and genetic triggers would force the birds to depart if they were already late.

## Methods

### Study area

**Fig. 1:**
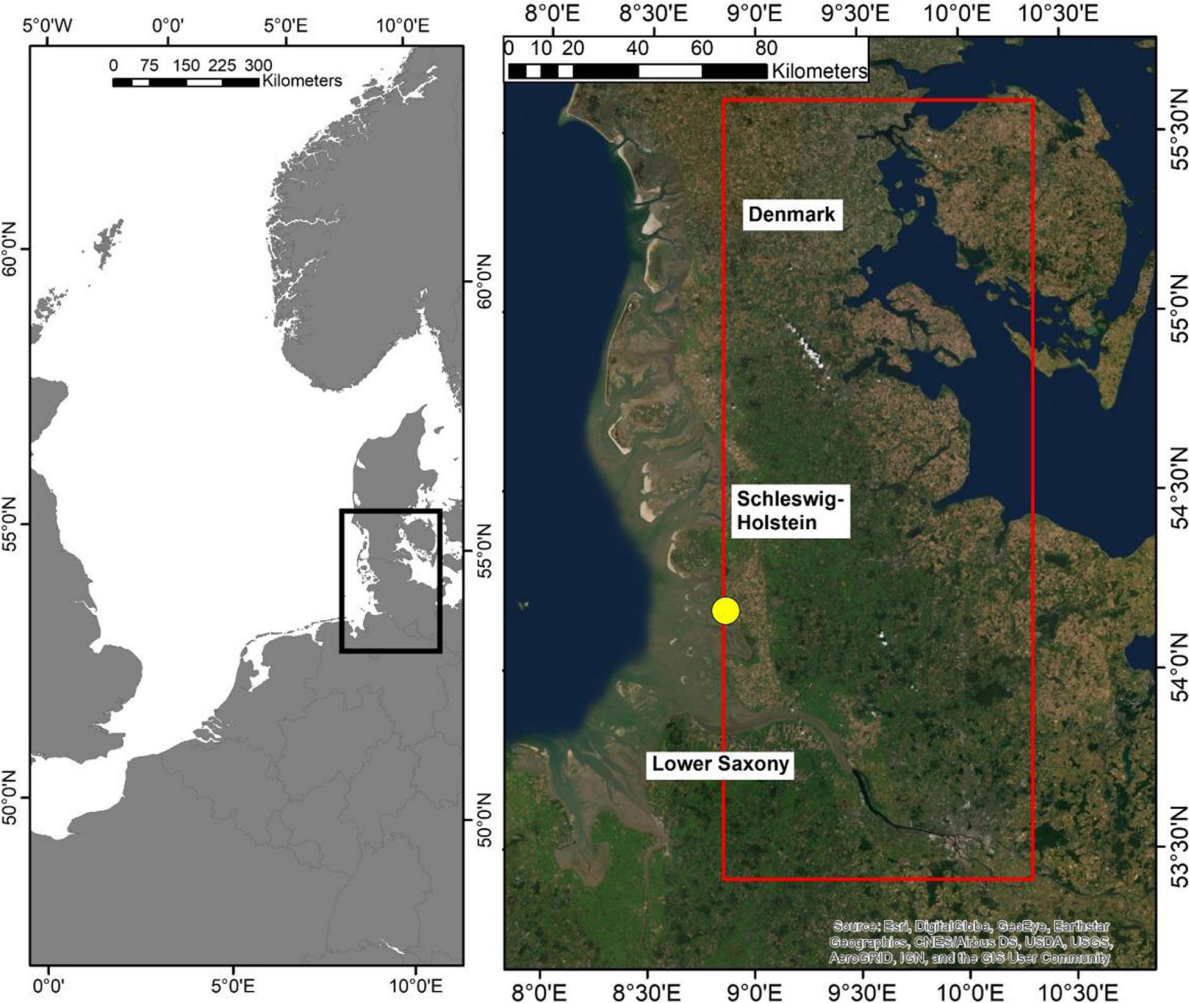
Left: location of study area in the southern part of the German Wadden Sea, south-eastern North Sea coast, indicated by black box. Right: study area for analyses of tailwind component flight speeds and flight altitudes (red box) and location of weather recording station (yellow dot). Satellite image: ESRI, DigitalGlobe, GeoEye, Earthstar Geographics, CNES/Airbus DS, USDA, USGS, AeroGRID, IGN, and the GIS User Community

Curlews were caught along the eastern Wadden Sea coast of the German federal states of Schleswig-Holstein and north-eastern Lower Saxony between 54°36′N and 53°42′N, and between 7°54′E and 8°54′E (Fig. 1). Meteorological parameters were recorded by the automatic recording station of the Research and Technology Centre, located in Büsum, federal state of Schleswig-Holstein (54°7′55″N; 8°52′37″E; yellow circle in Fig. 1). Flight speeds (using Doppler shift) and altitudes of tagged curlews were recorded simultaneously with GPS locations. Means of flight speed and altitude were calculated within an area stretching from the Wadden Sea coast to the Baltic Sea and from south Denmark to the northern part of the federal State of Lower Saxony (i.e. from the moment a bird entered the red box in Fig. 1 until it left the box).

### Catching of curlews and deployment of GPS tags

A total of 26 adult curlews were caught at their high-tide roosts using mist nets, between 2014 and 2020. Three of the GPS devices malfunctioned or the birds were depredated before departure from their wintering grounds, and data for 23 adult wintering curlews (11 females and 12 males) were therefore available for this study. Some curlews migrated multiple times before the device stopped working, and we were therefore able to record repeatability of departures and arrivals of the same individuals in subsequent years. All individuals were equipped with solar-powered GPS-Global System for Mobile Communications (GSM) data loggers attached by breast harnesses [31, 38]. The devices recorded time, date, geographical position, and flight speed at pre-programmed intervals of 1–15 min. Twelve individuals were equipped with “Skua” data loggers (Ecotone, Poland) weighing 17 g, and the other 11 individuals with OT-20 (3 individuals), OT-15 (7 individuals), and OT-10 (1 individual) data loggers, weighing 20, 15, and 10 g, respectively (Ornitela, Lithuania). Our study took place over a period of 7 years and we therefore aimed to use progressively lighter data loggers in line with technical developments (particularly logger weight reduction) over this time period. The mean body masses of female and male curlews were 957.7 ± 74.3 g and 827.8 ± 92.1 g, respectively (all values represent mean ± SD, unless otherwise specified). Even the heaviest data loggers used in this study therefore accounted for only about 2.4% of body mass, which was below the threshold of 3% suggested to avoid confounding effects of the devices [39]. The Skua devices only sent part of the data to a server via a GSM connection, which could then be downloaded directly, and the rest of the data were transmitted to base stations set up next to the high-tide roosts. The full dataset for the Skua devices was thus only obtained after the birds had returned to their wintering sites in the Wadden Sea. The highest temporal resolution achieved by these devices was 15 min. In contrast, the OT devices transmitted the whole dataset to an online portal via GSM network, and the recording intervals were programmed according to a flexible schedule, generally ranging from 1 to 15 min, based on the battery status of the device. We programmed “geofences” (i.e. defined areas in which the devices recorded data constantly in 1-min intervals) for all OT devices. The geofence covered the red box shown in Fig. 1, but excluded high-tide roosts, to save battery power. The high temporal resolution of the GPS fixes allowed flight height measurements to be derived within the red box area shown in Fig. 1, and also further east.

All data recorded by the GPS devices were stored in the online portal Movebank (www.movebank.org).

In addition to equipping each bird with a GPS device, all individuals were ringed and weighed, bill and wing lengths were measured, age was determined, and sex was determined by taking a breast feather for genetic sexing in the lab (Tauros Diagnostics, Berlin).

### Description and treatment of weather variables

Meteorological data at the wintering site (subsequently referred to as “local weather”) were recorded at 1-min intervals by an automatic recording station located at the Research and Technology Centre in Büsum (yellow dot in Fig. 1). The recorded parameters were: temperature (°C), wind speed and maximum wind speed (m/s), wind direction (degrees), precipitation (mm) global radiation (W/m^2^), air pressure (mbar), and air humidity (%) (all of which have been used before to predict departure decisions in songbirds [12, 35]). From global radiation we calculated a proxy for cloudiness by firstly fitting a generalized additive model (GAM) using the global radiation values as the outcome and the time of day as a smooth predictor – based on five pooled cloudless days. Secondly we used this model to calculate for all available days the deviations of global radiation from these predicted values. For all weather parameters, we computed the mean values of the 1-min recordings over the daylight hours of the departure/arrival day (i.e. from sunrise to sunset) of each individual curlew. The mean values were then related to the departure/arrival day of year (see Statistical analysis section below) and used to test for differences in weather conditions between departures and arrivals. To account for the circular nature of the wind direction, means were calculated as the direction of a circular vector using the R-package *circular* [40]. Wind-rose plots of the wind direction during the departure and arrival of curlews were created using the R-package *openair* [41].

Additionally, we used meteorological data collected on a larger scale (i.e. in an area east of − 3.30′ W, west of 25° E, south of 59° N and north of 50° N; subsequently referred to as “large-scale weather”). This data was obtained from the US National Centres for Environmental Prediction (NCEP) using the R-package RNCEP [42] and consisted of temperature, air pressure, the northward/southward wind component (u) and the eastward/westward wind component (v) – all variables obtained at the level of the earth’s/sea’s surface.

### Statistical analysis

We visualized the GPS data for each curlew using the Geographical Information System ArcGIS (version 10.3) [43]. The time (UTC) and day of the year at which the birds departed their wintering grounds heading north-east towards their breeding sites were determined. This departure was evident from the GPS tracks, and was always associated with a clear increase in flight speed (and flight heights in OT devices). The time and day of year of arrival at the wintering grounds was determined in a similar manner.

Departure and arrival dates were related to linear distance from the likely breeding area which was identified in GIS (white triangles in Fig. 4). It could be distinguished from stop-over locations by being the most distant point from the wintering location, and at the same time as a location where the birds stayed for several weeks (mean stay at breeding site: 55 days; range: 47–62 days; i.e. more than at each stop-over location) exhibiting non-directional movements. The approximate coordinate of the nest site was calculated by computing the mean geographical position of all positions in the potential breeding site. Subsequently, the linear distance between this position and the wintering site was calculated in GIS.

Departure dates were related to the linear and flown distances (calculated in GIS) to the first stop (red circles in Fig. 4), the flight time to the first stop, and the duration of the first stop. The same was applied for the arrival dates using the last stop before the wintering site (orange circles in Fig. 4). The mean locations of the nearest stops to the wintering sites were calculated in the same way as for the breeding site. In some cases, birds migrated from their wintering sites in the Wadden Sea to other areas (always < 30 km distance), probably to join other birds shortly prior to departure. These locations were not regarded as first stop-over events, and the departure from the last site in the Wadden Sea was used for the analyses instead.

Lastly, we computed the relative deviation between the flown and linear distances (%) as an indicator of the curvature of the flight track (i.e. the deviation of the actual flight track from a straight line). This was expected to increase during headwind conditions, because birds might try to avoid headwinds by choosing different flight angles relative to the wind direction (i.e. the actual flight line may start to meander). Only flight tracks with log-intervals of ≤5 min were used for this to keep the flight tracks comparable.

We determined the mean departure direction of each individual across the red box shown in Fig. 1 and related it to the mean recorded wind speed and wind direction to compute the tailwind component (TWC). This is known to have a significant impact on the migration speed of birds [32, 33, 44], and was therefore expected to affect the departure and arrival decisions of the curlews. According to [45], we used the following formula: TWC = *v* × cos *x*; where *v* is the wind speed in ms^− 1^ and *x* is the angular deviation between the opposite flight direction of the curlew (i.e., tailwind direction) and the wind direction (in degrees). In addition to using TWC as an additional predictor of departure and arrival decisions, we also related it to mean flight speed to demonstrate if the birds were able to increase their speed during tailwind conditions, and to mean flight height within the red box in Fig. 1.

All statistical analyses were carried out using the open source software R, version 3.6.3 [46]. Plots were visualized using the R package *ggplot2* [47]. All tests were considered significant at a level of *p* < 0.05, except the GLMM regressions following the LASSO approach for which we applied a correction for the alpha inflation (for explanation see below). For LASSO analysis, we used the R package glmnet [48], all other regressions were performed using the gamm() function [49, 50] using the R package *mgcv* [51]. Since no smooth terms have been considered, a generalized linear mixed model (GLMM) instead of a GAMM has actually been fitted. Here, individual was included as a random intercept in the GLMMs to avoid pseudo-replication due to multiple observations of the same individual. For each model, we selected an appropriate probability distribution for the variable of interest; if different probability distributions were reasonable (e.g. in the case of possibly overdispersed count data), we selected the most appropriate distribution based on the Akaike Information Criterion [52], e.g. comparing the poisson-, negative-binomial- and the tweedie-distribution.

We related the departure day to the local and large-scale meteorological data to see if departure decisions were affected by the weather. We considered the meteorological data under four conditions, including (1) the mean local weather conditions at the wintering site on the day of departure contrasted with the mean local weather conditions on the day prior to departure. (2) The same was done for the large-scale weather conditions. (3) The mean local weather conditions on the day of departure contrasted with the same measure on the same date for the average of the 4 previous years to determine if the curlews experienced suboptimal meteorological conditions compared with the average conditions on similar dates. (4) The same was done for the large-scale weather conditions. In all cases, the meteorological conditions were compared by dividing the mean weather data at the departure day by the mean conditions at the preceding day (or 4 preceding years, as applicable). If the meteorological variable of interest had a zero value, the difference was calculated instead of the quotient. For local weather data, variables have always been averaged from sunrise to sunset, for large-scale weather averages for 24 h have been used.

In addition to the above meteorological parameters, we also used the following additional predictors to model the departure decision (defined by day of the year): number of migrations for each individual bird, sex of the individual, catching location in the Wadden Sea, year, time of day, breeding latitude, linear distance to breeding area, departure direction, TWC, time to first stop-over, duration of first stop-over, flown distance to first stop, and linear distance to first stop. For TWC, we used the two different combinations of local meteorological data given above. All other predictors were kept constant.

We therefore used a total of 38 predictors to model the departure decision (i.e. 16 local and 8 large-scale meteorological predictors contrasted with the preceding day and with the four proceeding years, 2 contrasted combinations of TWC, and 12 constant predictors related to the individual curlews or the first stop-over event). The same predictors were used to model the arrival of the birds in their wintering grounds.

The effects of the 38 predictors on day of the year (outcome variable) were tested using the least absolute shrinkage and selection operator (LASSO) [53, 54] technique for predictor selection. This technique is known to handle a large number of possible predictors without being prone to statistical problems e.g. compared with stepwise methods (c.f., below). Notably, LASSO has been combined with cross-validation to select promising predictors based on their predictive capacity [53, 54]. In contrast to the common stepwise methods (e.g., forward or backward selection procedures), LASSO-based results are not sensitive to the order of the performed tests [55, 56]. However, it is necessary to bear in mind that the chance of detecting a significant relationship between a predictor and the considered outcome variable increases with the number of investigated predictors. Therefore, the final GLMM-based results do not follow from an a priori model with appropriate type I error control, since predictors have been pre-selected before, increasing the risk to discover random correlations (“alpha-inflation”). This problem is related to the problem of type I error control during multiple testing [57, 58]. Similar to the procedure for multiple tests, we therefore reduce the critical alpha-level and only discuss final GLMM-related variables that show a value *p* < 0.01.

## Results

### Phenology of departures and arrivals

**Fig. 2:**
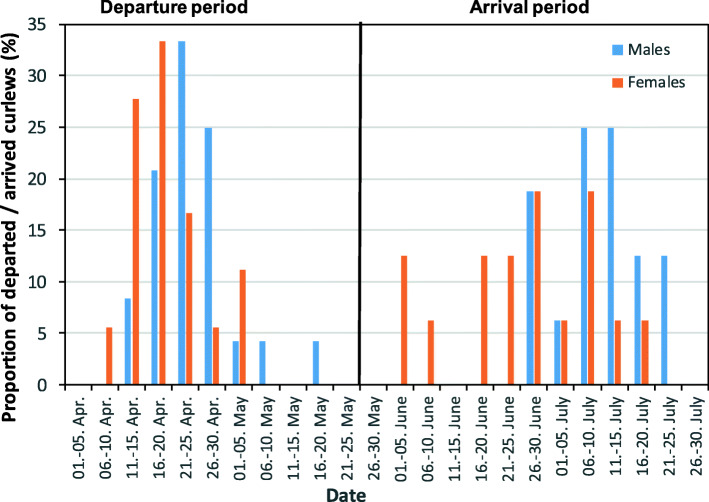
Proportion of curlews that departed from (left) and arrived at the wintering site (right) in relation to date

We recorded a total of 42 departures from and 33 arrivals at the wintering sites. The first curlew departed from the wintering grounds on April 7 and the last on May 16. Most individuals departed between mid- and late April (Fig. 2). Females tended to depart earlier, but the sex difference was not significant (GLMM: t = 1.23, df = 41, *p* = 0.23). The first curlews arrived at the wintering sites on June 3 and the last on July 24, with most birds arriving between mid-June and the end of July (Fig. 2). There was a clear but not significant tendency for females to arrive earlier than males (GLMM: t = 1.97, df = 31, *p* = 0.067; mean day of year females: 177.9 ± 13.1, mean day of year males: 189.9 ± 11.7).

**Fig. 3:**
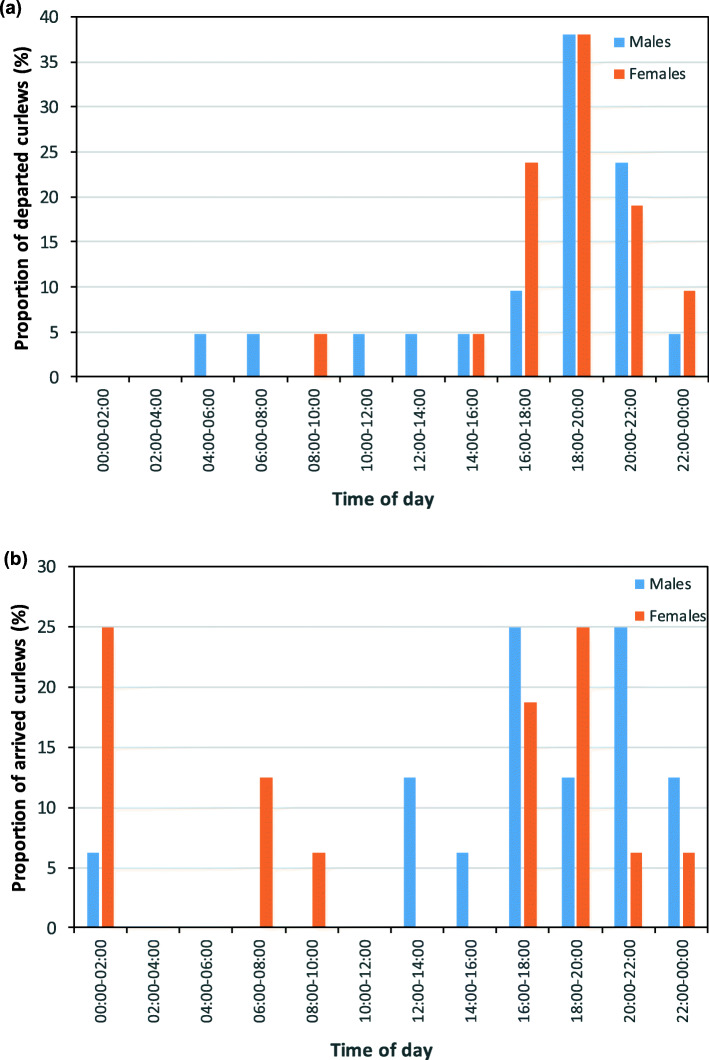
Proportion of curlews that departed from (**a**) and arrived at the wintering site (**b**) in relation to time of day (UTC)

Departures mainly occurred during the late evening, shortly before sunset, with no significant difference between the sexes (GLMM: t = − 0.29, df = 41, *p* = 0.78; Fig. 3a). The time of day for arrivals differed from that for departures (Table 1, Suppl. 1a), with significantly more arrivals during the nighttime and also occasionally during daytime. Similar to departing curlews, there were no differences between females and males in the timing of arrivals (GLMM: t = 1.7, df = 31, *p* = 0.09; Fig. 3b).

**Table 1:** GLMMs comparing migration parameters, and wind variables between curlews departing from and arriving at wintering sites. Boxplots illustrating the differences are shown in Supplement 1

	Estimate Std.	Error	t-value	*p*-value
Time of day	−3.08	1.32	−2.33	**0.023**
Rel. diff. between flown and linear distance to nearest stop	−2.06	1.75	−1.8	0.244
Linear distance to nearest stop	− 432.4	106.2	−4.07	**< 0.001**
Time to nearest stop	−189.47	118.16	−1.6	0.114
Duration of nearest stop	258.7	433.5	0.6	0.553
Mean flight speed	−18.53	4.12	−4.49	**< 0.001**
Tail wind component	−0.33	0.67	−0.5	0.618
Mean flight altitude	− 930.2	164.9	−5.64	**< 0.001**
Mean wind speed	−1.29	0.45	−2.89	**0.005**
Mean wind direction	−2.2	27.43	−0.08	0.936

GLMM outputs are illustrated in Suppl. 1

*Estimate Std* estimated standard deviation

### GPS tracks of curlews and relationships with nearest stop-over sites

**Fig. 4:**
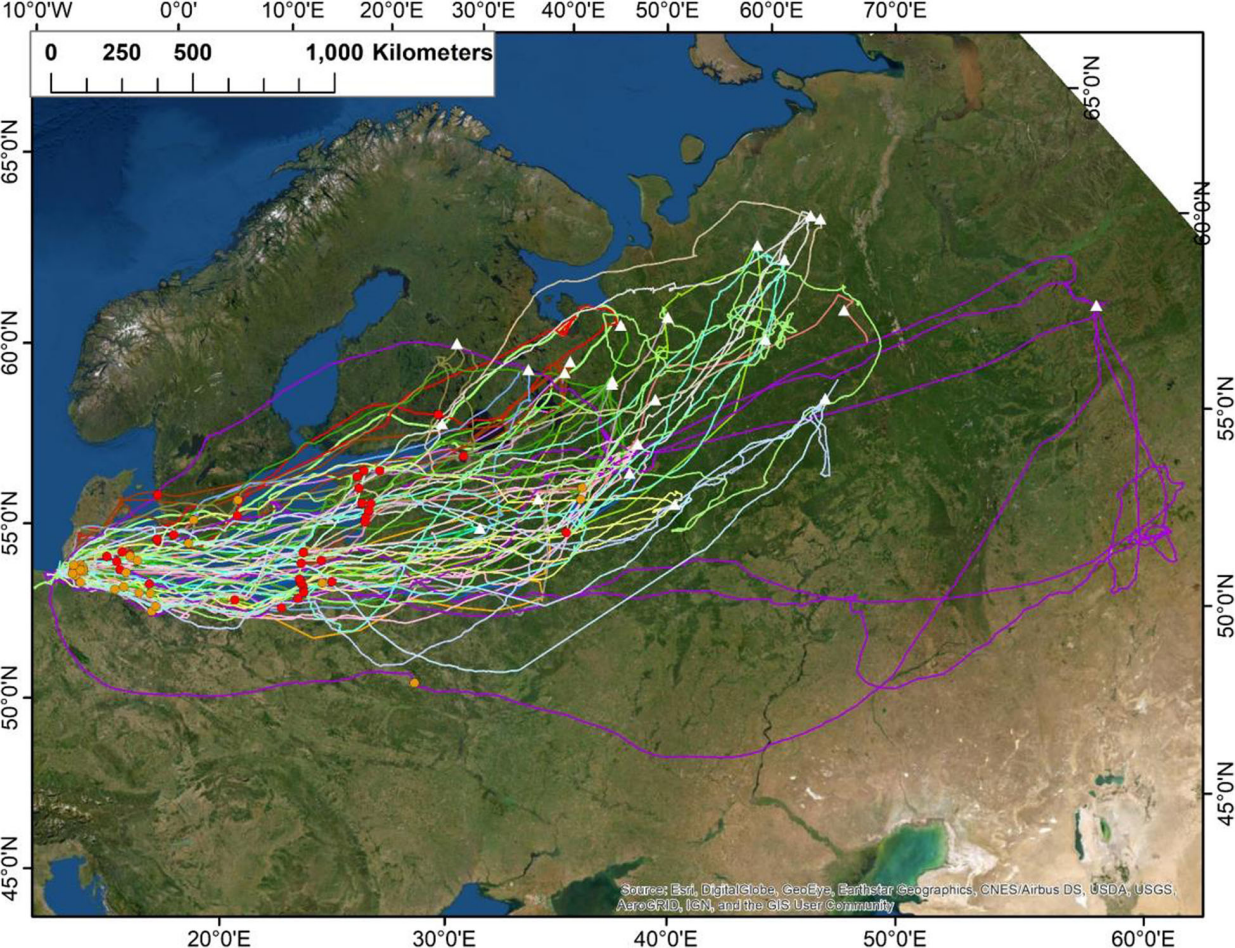
Flight tracks (*n* = 41) of 23 curlews (unique colour of each individual) between their wintering sites in the Wadden Sea and their breeding sites (white triangles). Red (northbound spring migration) and orange dots (southbound autumn migration) indicate location of the first and last stop-over sites, respectively. Satellite image: see Fig. 1

After their departure from the wintering grounds in the Wadden Sea, all curlews headed towards their breeding sites, which were located exclusively in north-western Russia (except for one individual that bred in Finland; see white triangles in Fig. 4). The most distant breeding site, a location east of the Ural Mountains, was 3840 km from the wintering site. The mean linear distance of all flight tracks was 2339 ± 612 km. The relative differences between the flown and linear distances to the breeding sites were similar for curlews arriving at and departing from the wintering grounds (Table 1; Suppl. 1b).

The mean linear distance between the nearest stop-over site and the wintering site for departing curlews was 775.8 ± 376 km, which represents 33.2% of the linear distance to their breeding sites (i.e. birds performed about 1/3 of their overall migration during their first migration bout). Curlews selected a straight flight path to reach their first stop-over site, with the distance flown on average only 45.7 km longer than the linear distance (5.6%).

During both, spring and autumn migration, many curlews crossed the Baltic Sea nonstop, while others stopped over on the Danish islands or the southern Baltic Sea coast (see red circles in Fig. 4). The nearest stop-over site for arriving curlews during autumn migration was significantly closer to the wintering site than that for departing curlews (mean: 342.4 km; Table 1; Suppl. 1c), but the distance was highly variable (± 503.1 km). In contrast, both time to the nearest stop-over site and duration of the nearest stop were similar for departing and arriving curlews (Table 1; Suppl. 1d, e).

Eventually, there was no significant relationship between flight time to the nearest stop-over and stop-over duration during spring and autumn migration.

### Flight speed and flight height in relation to tailwind component

**Fig. 5:**
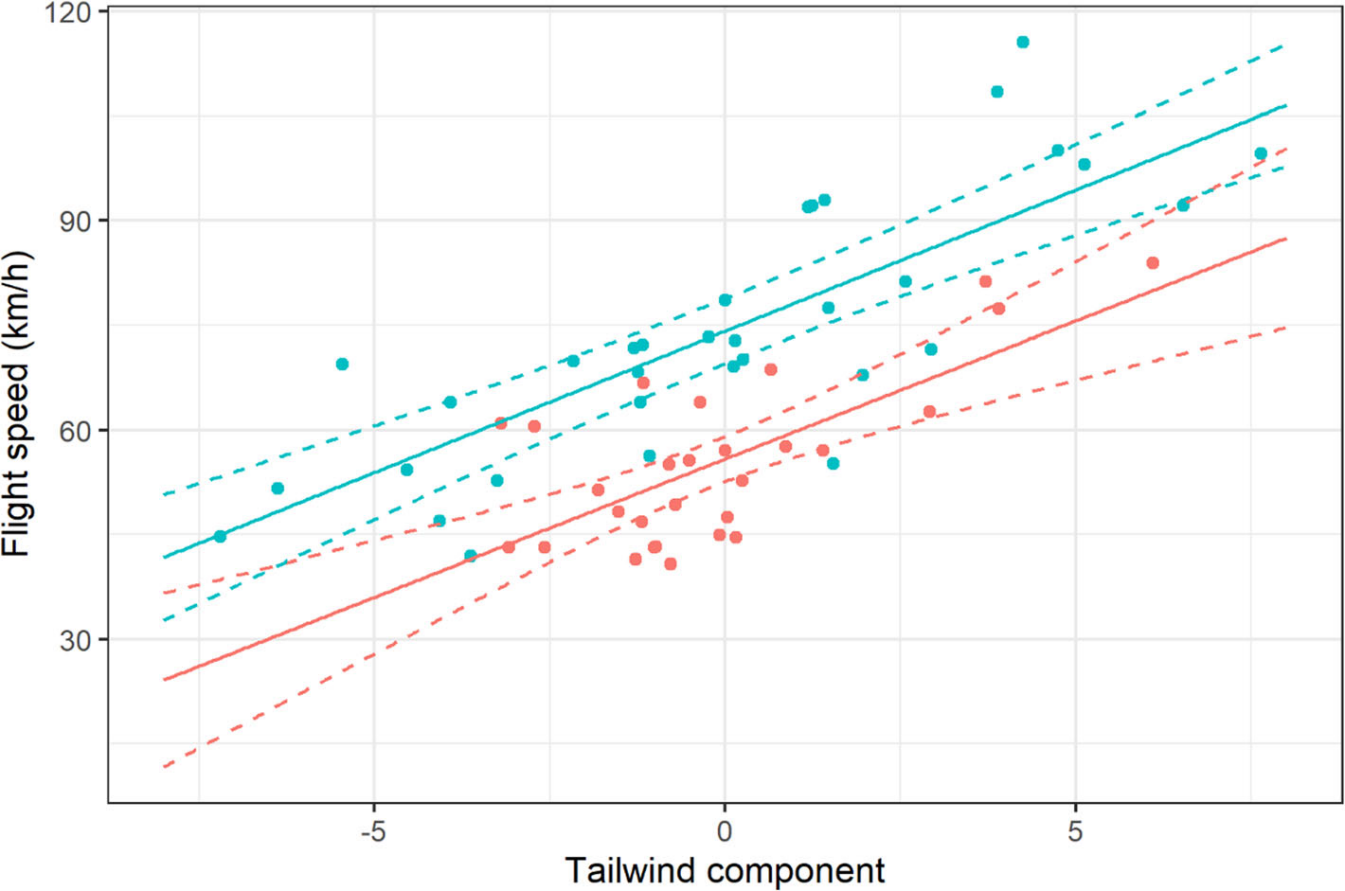
Flight speed of curlews departing from (blue) and arriving to (red) wintering site in relation to tailwind component. Solid line: model curve; dashed lines: 95% confidence intervals

The flight speeds of curlews both departing from (GLMM: t = 8.42, df = 32, *p* < 0.001) and arriving at (GLMM: t = 5.07, df = 27, *p* < 0.001) wintering grounds were positively and significantly related to TWC (Fig. 5), suggesting that birds were able to increase their migration speeds with wind assistance. There was no difference in the TWC relationships between departing and arriving curlews (Table 1; Suppl. 1f). Interestingly, flight speeds during departure were higher (mean: 73.8 ± 18.5 km/h, range: 41.9–115.6 km/h) than speeds during arrival at wintering grounds (mean: 55.3 ± 11.9 km/h, range: 40.8–84.0 km/h), but the 95% confidence intervals on these relationships overlapped only slightly (Fig. 5; see also Table 1; Suppl. 1 g).

**Fig. 6:**
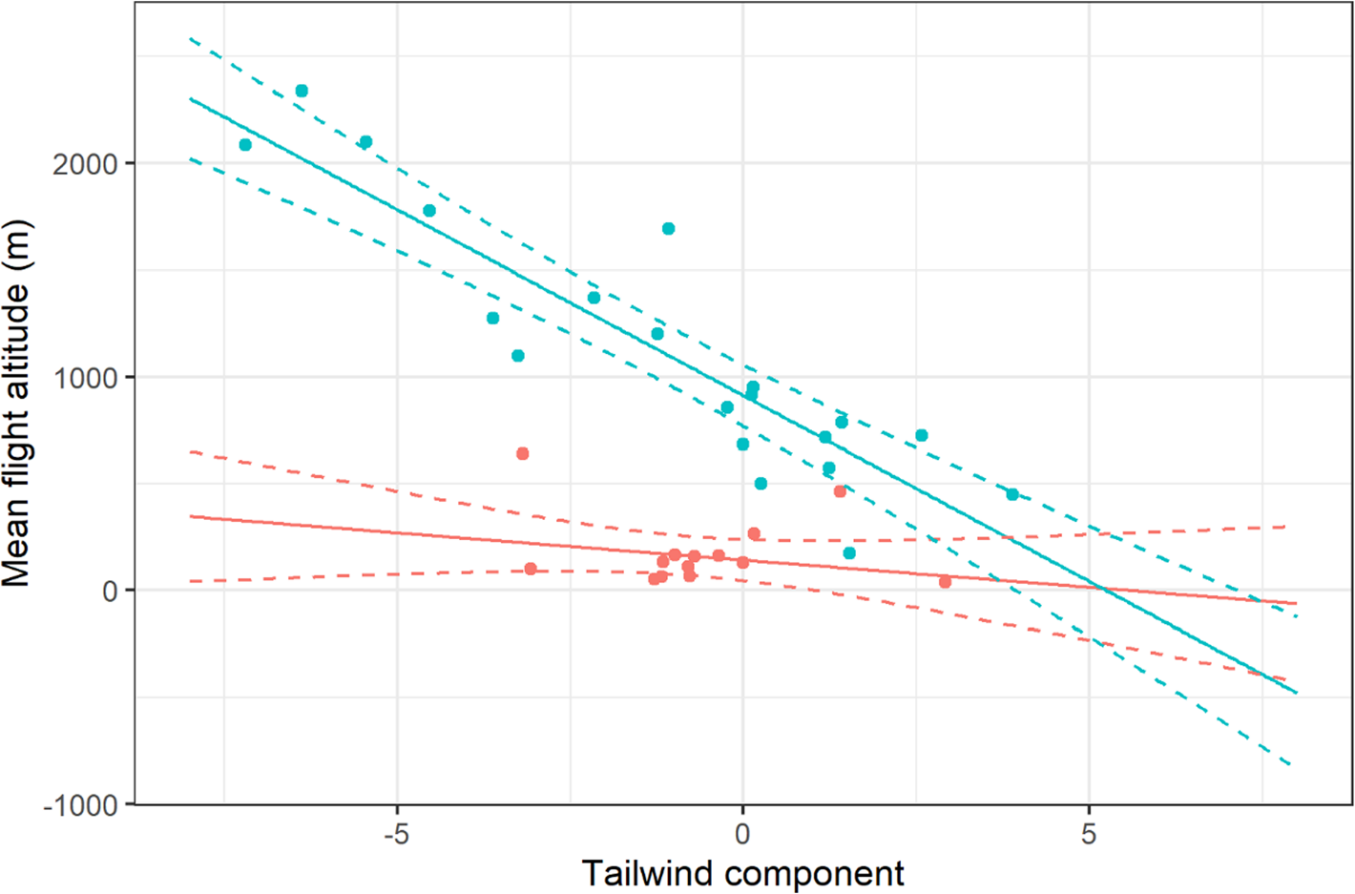
Flight altitudes of curlews departing (blue) and arriving to (red) wintering site in relation to tailwind component. Solid line: model curve; dashed lines: 95% confidence intervals

Curlews departed from their wintering sites at significantly higher altitudes during headwind compared with tailwind conditions (GLMM: t = − 9.52, df = 19, *p* < 0.001), but there was no significant relationship between TWC and flight altitude in curlews arriving to wintering sites (Fig. 6; GLMM: t = − 1.25, df = 13, *p* = 0.25). As for flight speeds, flight altitudes were significantly higher and more variable during departure (mean: 1113.3 ± 592.0 m, range: 175.2–2337.7 m) compared with during arrival at wintering grounds (mean: 182.3 ± 164.4 m, range: 37.0–639.2 m) (Table 1; Suppl. 1 h).

There was no significant relationship between TWC and the relative difference between the flown and linear distances, indicating that the straightness of the flight path was not impacted by wind conditions. Finally, there was also no correlation between TWC and migration distance to the first stop and no correlation between TWC and stop-over duration during spring and autumn migration, suggesting that headwind conditions had no effect on flight distances and length of the first stop-over.

### Departure/arrival decisions

**Fig. 7:**
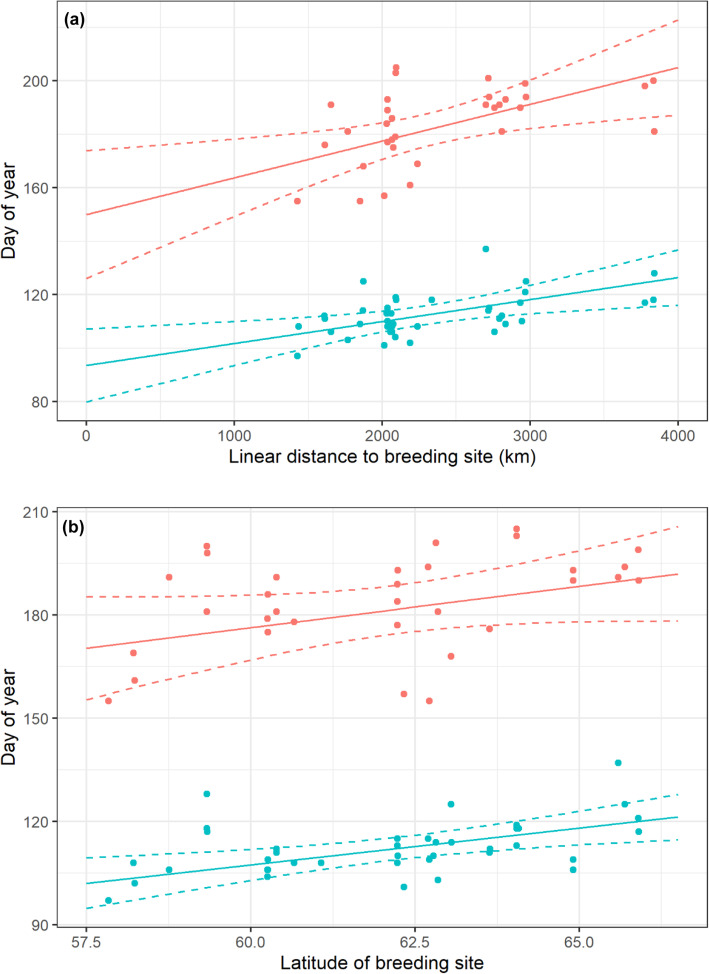
Day of year of curlews departing (blue) and arriving to (red) wintering site in relation to linear distance to the breeding site (**a**) and to breeding site latitude (**b**). Solid line: model curve; dashed lines: 95% confidence intervals

**Fig. 8:**
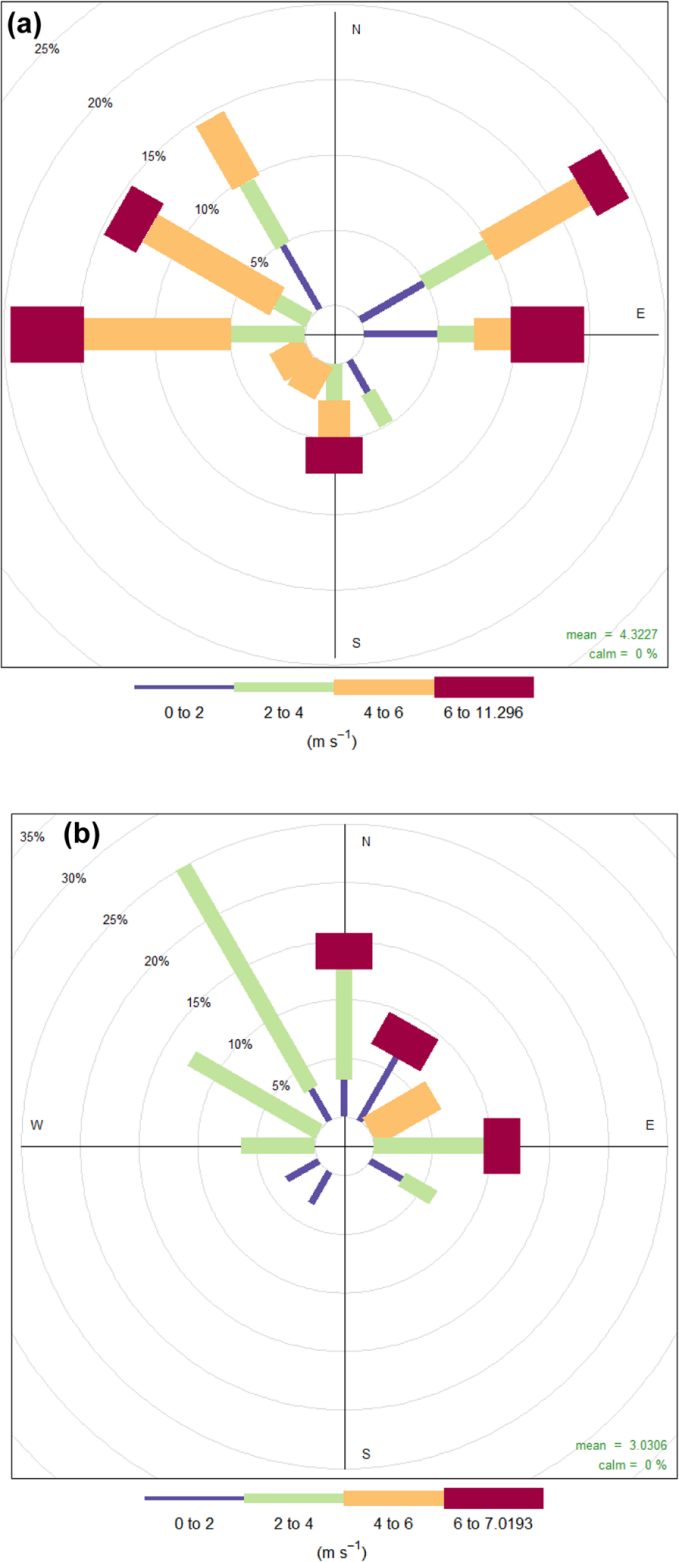
Frequency distribution of mean wind directions and wind forces (ms^− 1^) in the period from sunrise to sunset during day of departure from (**a**) and arrival to (**b**) wintering site

The LASSO + GLMM analyses showed that only two of the 38 predictors significantly influenced the departure day of curlews: curlews departed significantly later with increasing linear distance to their breeding sites (Fig. 7a; GLMM: t = 2.99, df = 37, *p* < 0.01) and when breeding at higher latitudes (Fig. 7b; GLMM: t = 3.61, df = 37, *p* < 0.01). Large-scale temperature at the time of departure from wintering grounds contrasted with the mean temperature at the same time of day, and day of year 4 years prior to departure remained a predictor in the final model, but had no significant impact on departure day when considering the alpha-level corrected significance level of *p* < 0.01 (GLMM: t = − 2.74, df = 37, *p* = 0.02). Interestingly, LASSO + GLMM analysis did not select any other local or large-scale meteorological predictors, bird-related variables, or variables associated with the nearest stop-over event. Mean wind direction and wind force on the day of departure were highly variable (Fig. 8a), which explains the absence of any significant relationships with day of departure.

According to LASSO + GLMM analysis, large-scale temperature contrasted with the mean temperature at the same time of day, and day of year 4 years prior to departure was the only predictor that remained in the final model for birds arriving at the wintering grounds. The higher the large-scale temperature (compared with the mean temperature of the preceding 4 years) the earlier the birds arrived back at their wintering grounds (GLMM: t = − 3.83, df = 37, *p* < 0.01). In the large-scale weather data, the mean vector of the northern/southern wind direction contrasted with the mean wind direction of the preceding day remained in the final model. However, when considering the alpha-level corrected significance level of *p* < 0.01 it had no significant impact on arrival day (GLMM: t = 2.81, df = 37, *p* = 0.02). The same was true for linear distance to breeding area (GLMM: t = 1.71, df = 37, *p* = 0.11).

As for departures, wind direction and force were highly variable (Fig. 8b) for birds arriving at their wintering grounds. Wind force (but not wind direction) differed significantly between departure and arrival flights (Table 1; Suppl. 1i, j). During arrival at wintering grounds, most of the higher wind forces were associated with north-easterly winds (Fig. 8b), which might have assisted some returning curlews, but eventually had no significant impact. This also led to a lack of any significant difference in tailwind conditions between departing and arriving curlews (Suppl. 1f).

In addition, there was no significant relationship between departure/arrival date and stop-over duration, indicating that curlews that migrated later did not have shorter stop-overs.

### Repeatability

**Fig. 9:**
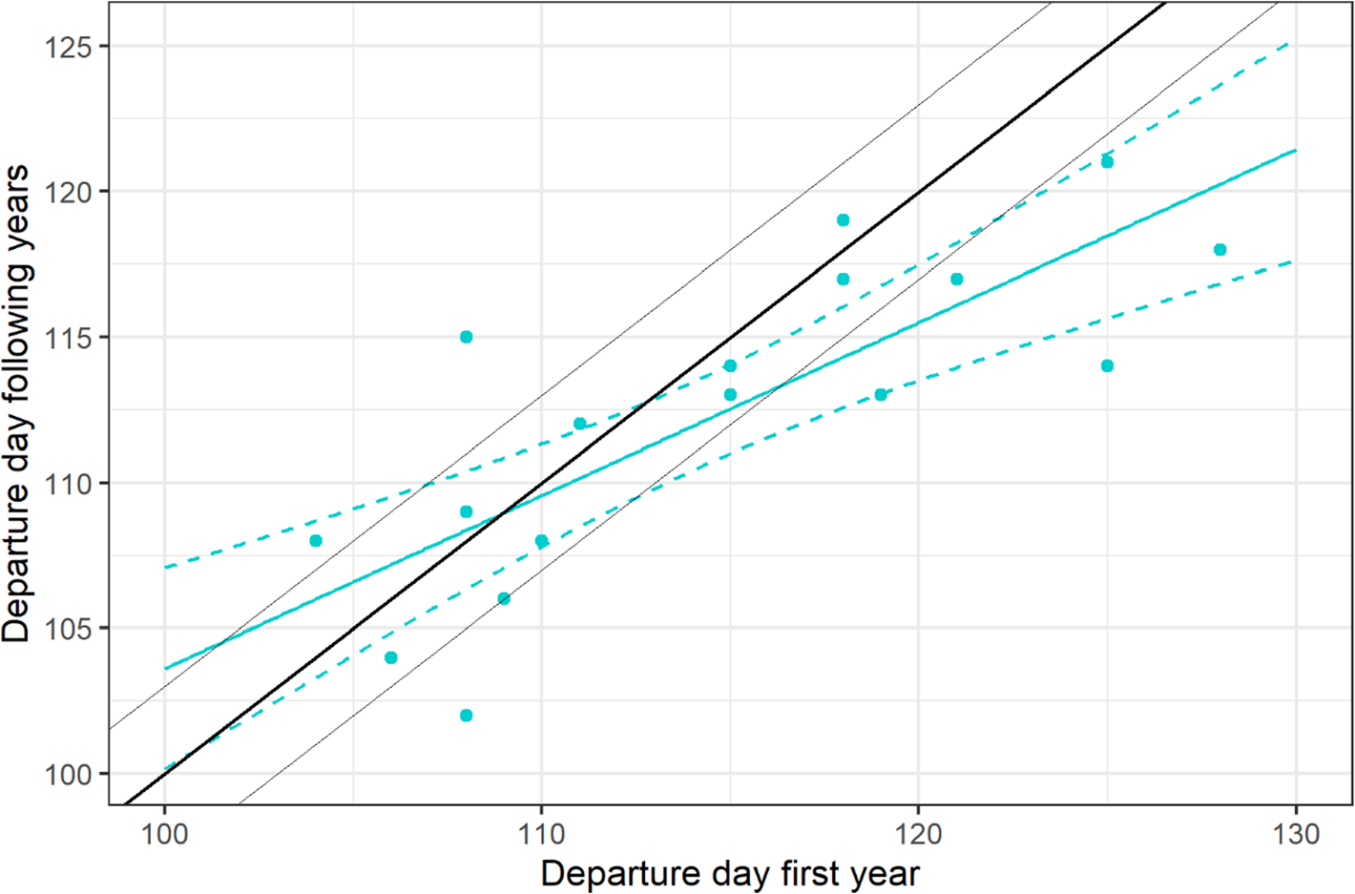
Departure days in successive years. Blue solid line: model curve; dashed lines: 95% confidence intervals, bold black line indicates 1:1 relationship, thin black lines depict departure days from wintering site 3 days before or after this

Among the 42 curlews with departure information, we recorded multiple departures from and arrivals at wintering grounds in subsequent years for 12 individual birds (departures: 8 individuals tracked for 2 years, 2 individuals for 3 years, 1 individual for 4 years and 1 individual for 5 years; arrivals: 5 individuals tracked for 2 years, 3 individuals for 3 years and 1 individual for 4 years). It was therefore possible to assess the repeatability of the departure and arrival day in the same individuals in different years. The individually-standardized mean absolute difference in departure days in subsequent years was only 3.68 ± 2.97 days (*n* = 19). However, the variability in arrival days of curlews returning to wintering grounds was more than twice as high (7.17 ± 4.83, *n* = 12). The departure days recorded in each individual’s first year were significantly related to the departure days in subsequent year(s) (Fig. 9; GLMM: t = 5.29, df = 18, *p* < 0.001), while there was no significant relationship for arrival dates (GLMM: t = − 0.5, df = 11, *p* = 0.62).

There was also high site fidelity in terms of the locations of breeding and wintering sites, but less fidelity for the location of the nearest stop-over sites (Fig. 4). The repeatability of flight directions was also high, with a mean variability of only 9.7 ± 8.2° for departures and 13.3 ± 10.2° for arrivals among subsequent years for the same individuals.

## Discussion

### Support for two concepts of bird migration

The current study aimed to disentangle three different concepts of bird migration (i.e. weather-related departure decisions, departure driven by environmental resources in destination areas, and genetic triggers). Given the absence of weather-related departure and the high repeatability of departure days of the same individuals in subsequent years, our results clearly support the concept of the existence of strong genetic triggers in our model species. The significant relationships between departure day and distance to breeding areas as well as breeding site latitude strongly suggests that the concept of resource availability in breeding sites is another decisive factor that determines the onset of migration in curlews.

### Low impact of weather effects

In contrast to other studies [12, 25, 27, 34] we found no evidence for local or large-scale weather phenomena as a migration trigger in curlews. The only exception was a higher than average large-scale temperature which was associated with earlier arrivals to wintering sites. The reason for this remains unclear. The whole autumn migration might have taken place faster during warm conditions, causing the birds to return earlier.

However, our data confirmed that flight speed increased with increasing TWC, in accordance with previous studies [6, 32]. Based on the clear benefit of faster flight speeds during tailwind conditions, this suggested that curlews would mainly select days with suitable tailwind conditions for their departure from (and arrival at) their wintering grounds in the Wadden Sea which was, however, not evident in our results. This is in accord with observations of curlews in China [59], and of the closely related whimbrel (*Numenius phaeopus islandicus*) [60], which also demonstrated no significant influence of wind force or wind direction.

Although weather parameters had no impact on departure/arrival decisions, we found a significant negative correlation between TWC and flight altitude in accordance with our second hypothesis. This clearly suggested that curlews tried to find more favourable wind conditions at higher altitudes if they encountered headwinds at lower altitudes as recorded in other shorebirds before [61]. In temperate latitudes, the prevailing westerly wind conditions in the higher air layers suggest wind assistance when ascending [62]. This behaviour has previously been recorded using radar techniques for nocturnal songbird migrants [35], as well as for diurnal long-distance migrants [26]. Dokter et al. [63] found intensive songbird migration in air layers up to 3 km altitude in temperate regions when birds encountered headwind conditions close to the surface. The authors demonstrated that migrating birds benefited from the wind conditions in higher air layers (that were only available in spring) by ascending. This might explain why there was no significant relationship between flight altitude and TWC in arriving curlews during their autumn migration. The current results clearly show that curlews depart (and stay) at lower altitudes when wind conditions close to the surface are beneficial, and use higher air layers during spring migration when they encounter headwinds.

Interestingly, curlews arrived at significantly slower flight speeds and lower altitudes compared with departing birds. Meteorological reasons for this can be excluded, given that the wind conditions and TWC were similar for departing and arriving individuals. One likely reason is that the linear distance to the nearest stop-over in arriving curlews was far smaller than for departing individuals, which might explain why departing curlews ascended to higher altitudes and had faster flight speeds compared with arriving birds.

### Seasonal timing of migration according to resource availability

Our data clearly supported our fourth hypothesis, i.e. the concept that the onset of migration was linked to factors related to the breeding sites (e.g. nest-site availability, resource access), given that distance to the breeding site and breeding site latitude were the single (highly significant) predictors affecting the day of departure in our LASSO analyses. An early arrival after snow melt will enable the curlews to establish their breeding territory and start their breeding activities in time to be ready for arthropod emergence. Previous studies demonstrated that arthropod densities in Arctic and sub-Arctic breeding grounds peaked shortly after snowmelt, resulting in higher chick growth rates if birds started nesting early [14, 15]. With respect to our study, this likely explains why curlews that breed further from their wintering grounds and in higher latitudes (e.g. in the north-eastern parts of Russia in this study) might delay their migration to ensure that they encounter optimal resources. Similarly, a previous study on curlews wintering in Britain [23] showed that colour-ringed curlews breeding in Fennoscandia departed their wintering site later than birds breeding further west. Although this study dealt with a different sub-population, the results were in agreement with the patterns found in the current study.

We found that curlews wintering in the Wadden Sea departed within a one-month time window (i.e. mostly between mid-April and mid-May). This contrasted with birds wintering in south-west Britain, which had already started to depart during February and March [23]. However, in contrast to curlews wintering in Britain and breeding in north-western Europe [23], all but one of the curlews in the present study bred in Russia, i.e. much further east. The more condensed departure window in curlews in our study might thus reflect the shorter window of opportunity in birds breeding in higher latitudes.

The main window of arrival of birds in the Wadden Sea was late June to mid-July, which is about 2 weeks later than reported for birds breeding in central or northern Europe [23]. Although desertion of offspring by females is common in shorebirds and has been shown for curlews [37], we found no sex differences in departure patterns, and only a non-significant tendency for females to arrive earlier (not in accordance with our fourth hypothesis). The reason for this is unclear. It is possible that some birds failed to rear chicks successfully, leading to the earlier arrival of at least some males.

### Diurnal patterns of migration start

Departures of songbirds usually occur at night and around sunset [17, 25]. Similarly, many long-distance shorebirds depart at night to take advantage of favourable atmospheric factors and to calibrate their orientation systems before they start in the evening hours [64, 65]. Our results support these patterns, with more curlews departing during the early evening or early nighttime. However, an earlier study of Eurasian curlews departing from a final pre-breeding stop-over site in China showed high variability in terms of the time of day for departures [59]. The reason for these different findings remains unclear.

### Correlations with first and last stop-overs

We expected that the departure day and TWC would be significantly related to the distance to the nearest stop-over site and the stop-over duration (in accordance with our third hypothesis); however, no such correlation was found. Curlews did not stage for shorter periods if they departed later, nor did they stage for longer if they encountered headwind conditions during the first part of their migration to allow more time for re-fuelling. This finding is in accordance with studies of songbirds, which likewise showed no or only weak relationships [66, 67] between TWC and distance to nearest stopover and duration of stopover. Similarly, no impacts of wind conditions on stop-over patterns were found for whimbrels wintering in west Africa and stopping over in Ireland or the UK on their way to Icelandic breeding grounds during spring migration. In contrast to our study, however, whimbrels tended to skip a potential stop-over when they departed later [60].

### High repeatability in departure patterns suggest genetic triggers

To the best of our knowledge, the current study is among the first to record high-resolution GPS-movement data in birds across consecutive years which also allows to identify repeated diurnal patterns of departure and subsequent movements including flight altitudes, flight speeds and repeatability of departure decisions. Studies on repeated individual-based migration patterns across consecutive years are scarce (but see [19] for a study using GPS-tags, [20, 21, 68] using geolocators and [22] using satellite tags). We found a high degree of repeatability particularly in curlew departure but also in arrival dates from / at wintering sites for the same individuals across subsequent years. The slightly higher variability in arrival dates among individuals is likely caused by varying breeding success (as successful breeders are thought to return to their wintering sites later [20, 37]). Given the absence of clear relationships between departure decision and wind/weather parameters, this high intraspecific repeatability clearly supports the concept of an internal genetic trigger (see also reviews in [16–18]), which seemed to play the most important role in departure and arrival decisions in our model species and therefore confirming our fifth hypothesis.

A recent review [18] presented evidence for genetic control of the timing of bird migration. However, the authors also found considerable individual variation in this genetic programme, as a result of interactions with environmental and social factors, and individual learning. Given the highly repeatable, conservative time pattern and lack of any relationships between departure date and weather parameters for our model species, the current results suggested that such intraspecific variation in the genetic programme may be very low for curlews. Our results thus provide robust support for the concept of an internal clock (i.e. a genetic control), responsible for timing bird migration [16, 17]. Intra-specific variation in departure decision was lower in curlews as compared to previous studies of other long-distant migrants [19–21] which suggests the existence of strong selective forces in our model species [19].

## Conclusions

We used data from long-term attachment of high-resolution GPS devices across multiple years to simultaneously explore the relative impacts of weather-related, time-driven, and genetically-induced migration decisions. In contrast to previous studies in different groups of bird species [9–12, 27], our results suggest that weather has only a minor effect on migration in curlews; if their individual departure day happens to coincide with headwind conditions, the birds simply account for this by flying at higher altitudes. However, further studies are needed to determine if curlews have knowledge of the wind conditions at higher altitudes when they encounter headwinds on the ground. Intraspecific variability in departure decisions was mainly driven by differences in the locations of the breeding sites, which underpins the importance of synchronising their departure with resource availability after the snowmelt in Arctic and sub-Arctic breeding sites. The low intra-individual variability in departure decisions in subsequent years, however, clearly suggests a strong genetic trigger regulating the timing of migration. Based on our results, we postulate that genetic triggers controlling the timing of migration are so pronounced in some bird species that extrinsic factors, such as weather, play only a minor role. Follow-up studies on closely related bird species as well as migration studies on immature curlews, ideally by tagging birds from the same clutch and their parents, will further help us to understand the genetic mechanisms triggering temporal migration patterns.

## Supplementary Information

**Additional file 1 **: **Supplement 1**. Differences in time, stop-over parameters, flight altitude, height, tailwind component, and two selected wind variables between departing and arriving curlews. For statistics see Table 1. Bold black dot: mean, black bars: 95% confidence intervals.

## Data Availability

The datasets generated during and / or analysed during the current study are available in the Movebank Data Repository, 10.5441/001/1.715k46g2 [69].

## Abbreviations

GPS: Global Positioning System
GSM: Global System for Mobile Communications
TWC: Tailwind component
