# Supplementary material for: Migrating curlews on schedule: departure and arrival patterns of a long-distance migrant depend on time and breeding location rather than on wind conditions
Source: Mov Ecol. 2021 Mar 17;9:9. doi: 10.1186/s40462-021-00252-y (PMC7967988; doi:10.1186/s40462-021-00252-y)
Supplement: Supplementary file 1 — Additional file 1 : Supplement 1. Differences in time, stop-over parameters, flight altitude, height, tailwind component, and two selected wind variables between departing and arriving curlews. For statistics see Table 1. Bold black dot: mean, black bars: 95% confidence intervals. [file 40462_2021_252_MOESM1_ESM.docx]

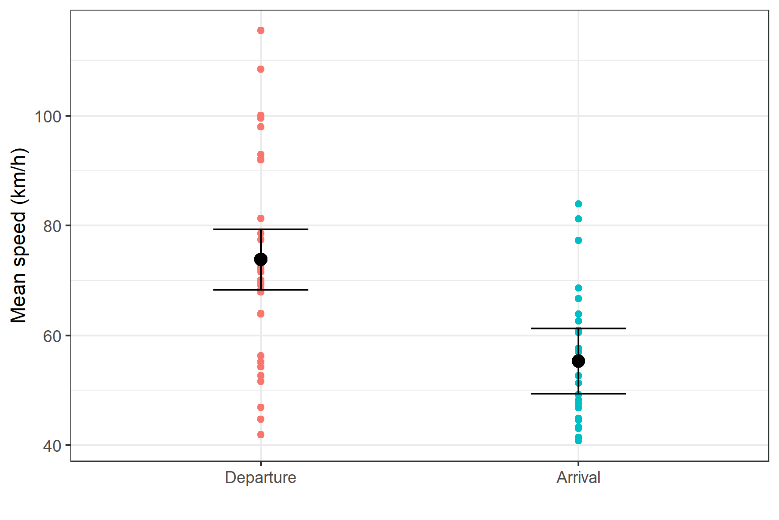

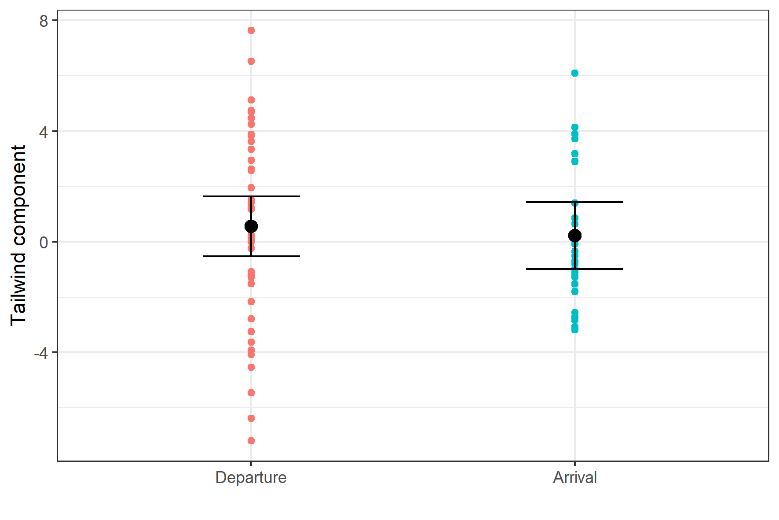

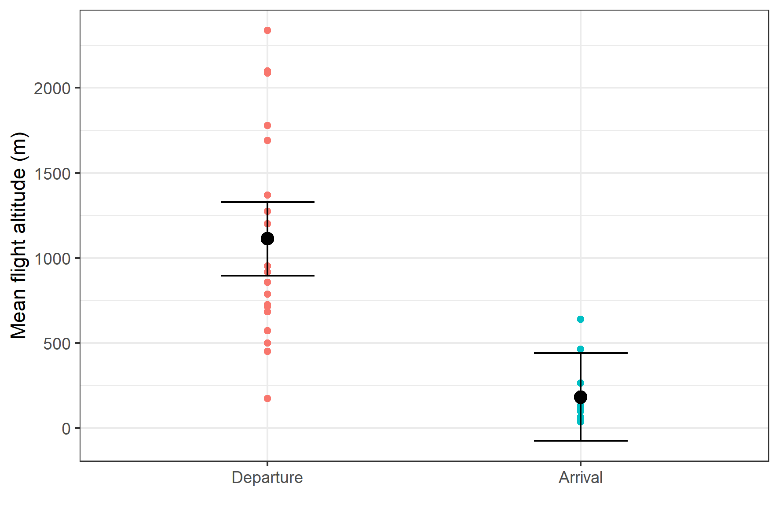

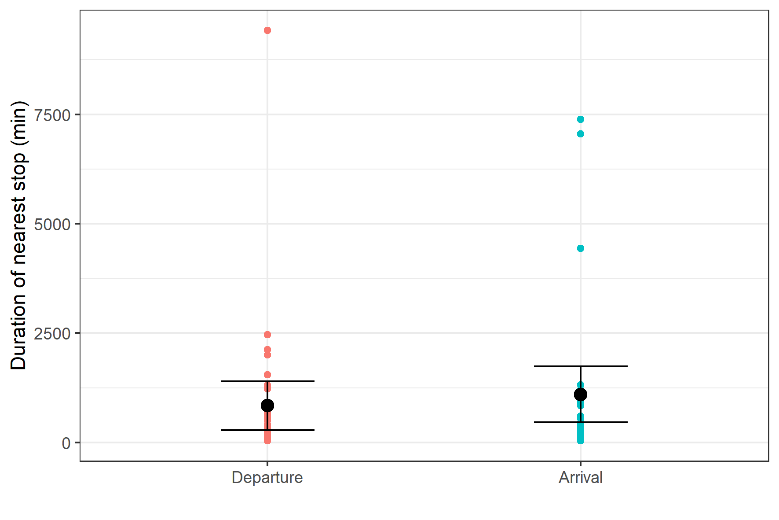

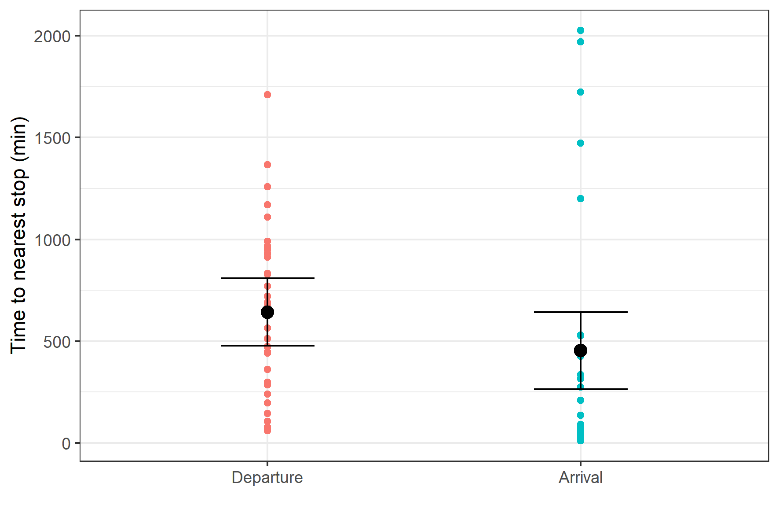

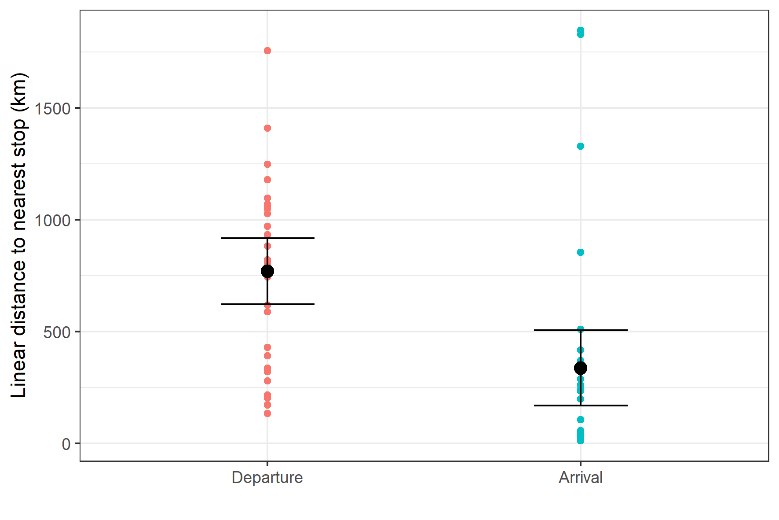

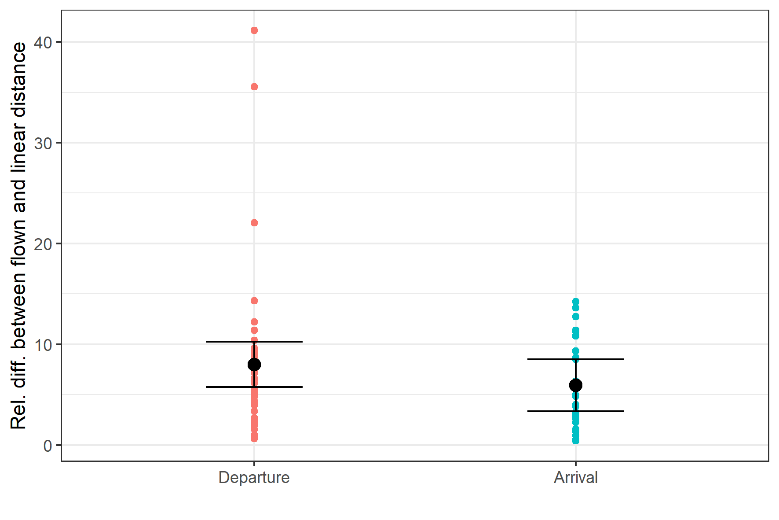

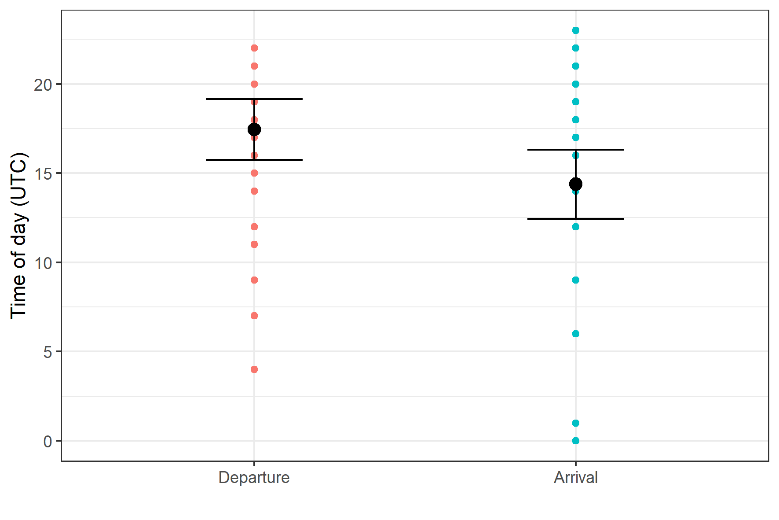
Supplement 1

(h)

(g)

(f)

(e)

(d)

(c)

(b)

(a)


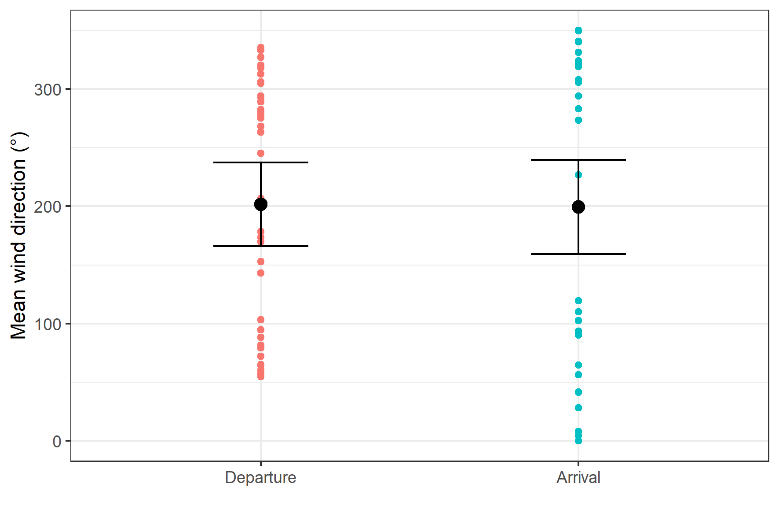

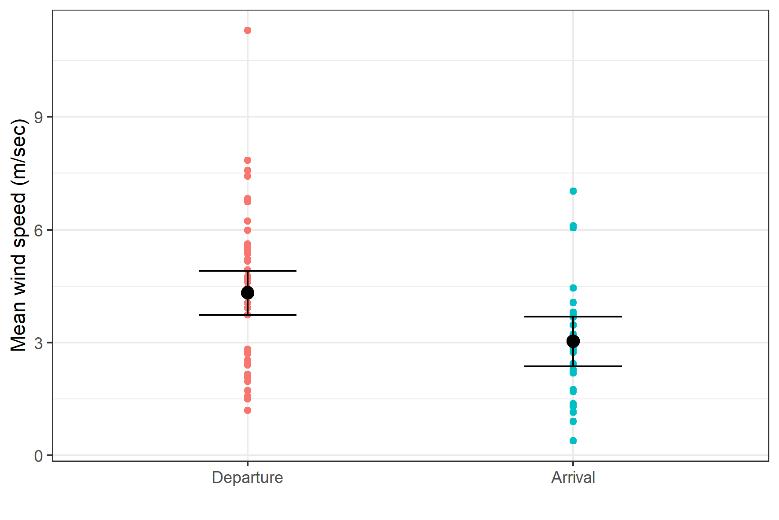
Supplement 1: Differences in time, stop-over parameters, flight altitude, height, tailwind component, and two selected wind variables between departing and arriving curlews. For statistics see Table 1. Bold black dot: mean, black bars: 95% confidence intervals.

(j)

(i)
